# Exposed Nerves

**Published:** 1872-06

**Authors:** 


*Exposed Nerves.*—Under the title of "Capping the Nerve Simplified," Dr. G. V. N. Relyea gives his method of treatment in the *Canada Journal of Dental Science.*

"I have within the last year or more adopted the following simple course when I found the nerve exposed, or when accidentally or intentionally uncovering it. Let me explain what I mean by intentionally uncovering a nerve. I hold that it would be perfect madness to suppose that a tooth that has pained for days, or perhaps weeks, from a diseased or inflamed nerve, can be successfully treated or the nerve tamed without a little 'blood letting.'

"The tooth being free from disease externally, which can readily be ascertained by the slightest pressure, the conclusion arrived at must be an abnormal condition internally, either suppuration or congestion of the nerve. If suppuration, the case in all probability is complicated, involving other parts, and will require a line of treatment not properly coming under this head, but which I will consider in a future article.

"If it proves to be inflammation, or even congestion of the nerve, the case is simple. I endeavor very gently to lift the dentine covering the engorged nerve, and the surplus blood being thus released from its imprisonment, will readily escape and the patient experience relief. I allow it to bleed freely for a minute, then apply a pledget of cotton saturated in carbolic acid. This I leave about five minutes, then remove and prepare my cavity. Should it continue to bleed while excavating, all the better. When I consider my cavity ready I apply more carbolic acid, and leave it while I get my gold and instruments in readiness. Next, I fold a piece of bibulous paper four thicknesses, saturate in carbolic acid, and cut about twice the size of the opening. The fingers will absorb the free acid, leaving it just sufficiently oily to cause to adhere to the walls of the cavity, If the cavity is small I carefully place my cap over the opening, put in sufficient gold to cover the floor of the cavity, which I gently press to its place, then wedge and finish up as usual. Should the cavity be large, I fill close to the opening, then place the cap in its destined bed and proceed with my filling. So successful have I been under this treatment that the wounding of a nerve gives me no concern, and I not unfrequently fill from one to three cavities the same day, in the manner before stated, and, indeed, sometimes for the same patient. Many of the teeth thus treated I never hear of again, and judging from those I got a chance to examine, I can say with safety, I do not lose one out of fifty teeth, though many of my patients are suffering when they come to the office, and they come fully determined to have their teeth extracted. If the plan I have endeavored to explain very briefly and imperfectly is followed and done with great care, the patients will leave the office free from pain, and thank you a thousand times (that is, if they have any gratitude, but some have none) for having relieved them from pain and saved their teeth."

